# From one incision to one port: The surgical technique and the evolution of segmentectomy in patients with pulmonary tuberculosis

**DOI:** 10.1371/journal.pone.0197283

**Published:** 2018-05-15

**Authors:** Yau-Lin Tseng, Chao-Chun Chang, Ying-Yuan Chen, Yi-Sheng Liu, Lili Cheng, Jia-Ming Chang, Ming-Ho Wu, Yi-Ting Yen

**Affiliations:** 1 Division of Thoracic Surgery, Department of Surgery, National Cheng Kung University Hospital, College of Medical College, National Cheng Kung University, Tainan, Taiwan; 2 Institute of Clinical Medicine, College of Medical College, National Cheng Kung University, Tainan, Taiwan; 3 Department of Diagnostic Radiology, National Cheng Kung University Hospital, College of Medical College, National Cheng Kung University, Tainan, Taiwan; 4 Division of Thoracic Surgery, Department of Surgery, Chia-Yi Christian Hospital, Chia-Yi, Taiwan; 5 Division of Thoracic Surgery, Department of Surgery, Tainan Municipal Hospital, Tainan, Taiwan; 6 Division of Trauma and Acute Care Surgery, Department of Surgery, National Cheng Kung University Hospital, College of Medical College, National Cheng Kung University, Tainan, Taiwan; Peking University People's Hospital, CHINA

## Abstract

**Objectives:**

We retrospectively reviewed the evolution of segmentectomy for pulmonary tuberculosis (TB) and the feasibility of multi- and single-incision video-assisted thoracoscopic segmentectomy.

**Methods:**

Of 348 patients undergoing surgery for TB, the medical records of 121 patients undergoing segmentectomy between January 1996 and November 2015 were reviewed. Clinical information and computed tomography (CT) image characteristics were investigated and analyzed.

**Results:**

Eighteen patients underwent direct or intended thoracotomy. Sixty-four underwent video-assisted thoracoscopic segmentectomy (VATS), including 53 multi-incision thoracoscopic segmentectomy (MITS), and 11 single-incision thoracoscopic segmentectomy (SITS). Thirty-nine were converted to thoracotomy. The intended thoracotomy group had more operative blood loss (*p* = 0.005) and hospital stay (*p* = 0.001) than the VATS group although the VATS group had higher grade of cavity (*p* = 0.007). The intended thoracotomy group did not differ from converted thoracotomy in operative time, blood loss, or hospital stay, and the grade of pleural thickening was higher in the converted thoracotomy group (*p* = 0.001). The converted thoracotomy group had more operative blood loss, hospital stay, and complication rate than the MITS group (*p* = 0.001, *p*<0.001, and *p* = 0.009, respectively). The MITS group had lower pleural thickening, peribronchial lymph node calcification, cavity, and tuberculoma grading than the converted thoracotomy group (*p*<0.001, *p* = 0.001, 0.001, and 0.017, respectively). The SITS group had lower grading in pleural thickening, peribronchial lymph node calcification, and aspergilloma grading than the converted thoracotomy group (*p* = 0.002, 0.010, and 0.031, respectively). Four patients in the intended thoracotomy group and seven in the converted thoracotomy group had complications compared with three patients in the MITS and two in the SITS group. Risk factors of conversion were pleural thickening and peribronchial lymph node calcification.

**Conclusion:**

Although segmentectomy is technically challenging in patients with pulmonary TB, it could be safely performed using MITS or SITS and should be attempted in selected patients. Its efficacy for medical treatment failure needs investigation.

## Introduction

Despite improvements in medications, therapeutic resection remains not only pivotal, but also technically challenging for treating the complications in pulmonary tuberculosis (TB). The rationale is to resect the damaged parenchyma complicated with hemoptysis, and lesions harboring actively replicating bacilli to reduce overall organism burden, and to remove the sites of compromised drug penetration [1–5]. For benign pulmonary diseases, the benefits of surgical intervention should always outweigh invasiveness and the potential loss of pulmonary function. Because anatomic distribution and parenchymal destruction are taken into consideration, segmentectomy should be attempted to effectively achieve disease control and preserve the lung parenchyma.

Since its introduction in the 1980s, video-assisted thoracoscopic surgery (VATS) has evolved and become the preferred approach for thoracic surgery over the past decade. Since 2007, when the VATS therapeutic resection was reimbursed by the National Health Insurance Administration in Taiwan, VATS has been our preferred method for both patients with malignancies, and those with complications of pulmonary tuberculosis (TB). VATS has been strongly recommended because of advantages, such as less wound pain, fewer pulmonary complications, and shorter hospital stay compared with thoracotomy [6–9]. Although pulmonary segmentectomy is more technically demanding than lobectomy, the safety, feasibility, and results of VATS for small peripheral lung cancer have been satisfactory compared with VATS lobectomy [10–14]. VATS segmentectomy should therefore be viewed as a surgical option, because patients with complications of pulmonary TB often present with localized disease extending from a segmental bronchus. Further, the fast recovery from surgery helps patients with active infection or relapse, continue the medical treatment. In addition to the conventional VATS, or multi-incision thoracoscopic surgery (MITS), we started to perform single-incision thoracoscopic surgery (single-incision VATS, or SITS) for anatomic resection in 2015. Literature regarding the feasibility and efficacy of segmentectomy for pulmonary TB, however, was still limited. We therefore reviewed our surgical results of patients with pulmonary TB undergoing segmentectomy and delineated the technical evolution and feasibility from thoracotomy to SITS, as well as the association with image characteristics.

## Material and methods

### Patient enrollment

Between January 1996 and November 2015, among the 348 patients who had surgery for pulmonary TB, the medical records of 121 patients who underwent segmentectomy for pulmonary TB were retrospectively reviewed. Informed consent was waived because the study was retrospective, and the review of medical records was approved by the Institutional Review Board of National Cheng Kung University Hospital (B-ER-100-213, B-ER-101-080). Clinical information was collected on age, sex, surgical indications, ultimate surgical approach and procedures, operative time, blood loss, length of hospital stay, complication, treatment outcome, and follow-up duration. Preoperative sputum yields were also investigated and recorded. Panel discussion among board-certified chest physicians specializing in pulmonary TB and thoracic surgeons under the surveillance of Center for Disease Control, Ministry of Health and Welfare, were routinely held and collaborated with Center for Infection Control in National Cheng Kung University Hospital. Surgical intervention was indicated in patients with hemoptysis or medically failed pulmonary TB. Patients with medically failed pulmonary TB included 3 groups: those who were sputum smear- or sputum culture-positive at 4 months or later after the initiation of anti-TB treatment, those with drug-resistant strain and radiographic cavitation, and those with unresolved radiographic lesion despite of culture-guided medication with disease relapse or high risk of disease relapse. Patients of high-risk treatment failure or disease relapse were those with pulmonary TB, diabetes mellitus, and a cavitary lesion in the lung on the image study. Patients were also considered of high risk if they had localized pulmonary TB and diabetes mellitus but not readily accessible to the appropriate medications, which needed to be specifically imported, such as kanamycin, streptomycin, capreomycin, terizidone, and clofazimine. Patients newly diagnosed with pulmonary TB, or those who underwent surgery due to treatment failure, were administered anti-TB medications for six additional months; those who underwent surgery due to multi-drug resistant TB (MDRTB) were administered anti-TB medications for at least 24 months after the sputum culture had become negative for TB.

### Classification and grading of image characteristics

The preoperative chest CT scans were reviewed by two board-certified radiologists, specializing in thoracic images, who were blinded to the patients’ identities and ultimate operative procedures. Abnormal findings identified on the chest CT scans were interpreted and classified as bulla, pleural thickening, peribronchial lymph node calcification, tuberculoma, cavity, aspergilloma, atelectasis, and bronchiectasis, with the radiographic criteria reported in Table 1 and our previous article [15].

**Table 1:** Definition and grading of image characteristics on chest computed tomographic scan in patients who underwent anatomical lung resection for pulmonary tuberculosis.

Image characteristics	Grading	Definition
Bulla	0	No such lesion
	1	Thin-walled air-containing space noted in only one lobe on the operated side
	2	Thin-walled air-containing space noted in more than one lobe on the operated side
Pleural thickening	0	No pleural thickening
	1	Pleural thickening in less than four 10-mm-thick sections or involving less than half the circumference on the operated side
	2	Pleural thickening in at least four 10-mm-thick sections, involving more than half the circumference on the operated side
Peribronchial lymph node calcification	0	No such lesion
	1	Single area of peribronchial lymph node calcification on the operated side
	2	Multiple areas of peribronchial lymph node calcification on the operated side
Tuberculoma	0	No such lesion
	1	Well-defined nodule or nodule with calcification under TB context in only one lobe on the operated side
	2	Well-defined nodule or nodule with calcification under TB context in more than one lobe on the operated side
Cavity	0	No such lesion
	1	Single thick-walled air-containing space on the operated side
	2	Multiple thick-walled air-containing spaces on the operated side
Aspergilloma	0	No such lesion
	1	Single lung lesion with “ball-in-hole” appearance noted on the operated side
	2	Multiple lung lesions with “ball-in-hole” appearance noted on the operated side
Atelectasis	0	No such lesion
	1	Decrease of lung volume in only one lobe on the operated side
	2	Decrease of lung volume in more than one lobe on the operated side
Bronchiectasis	0	No such lesion
	1	Dilated bronchial tree in only one lobe on the operated side
	2	Dilated bronchial tree in more than one lobe on the operated side

### Techniques

The patient was placed in the full lateral decubitus position under general anesthesia with selective one-lung ventilation. Before 2007, all the surgeries were done using direct thoracotomy. After 2007, all the surgeries were initiated with VATS (MITS and SITS), but were converted to thoracotomy if dense adhesion in the pleural space or around the hilar structures precluded VATS. A thoracostomy at the 6th intercostal space in the mid-axillary line was created for the flexible (EVE-L; Fujinon, Wayne, NJ, USA) or rigid (Karl Storz Endoscopy-America, Culver City, CA, USA) 30° thoracoscope through a 10.5-mm trocar. A utility thoracostomy of 3 to 5 cm at the third or fourth intercostal space was created and protected with a wound retractor (Alexis; Applied Medical, Rancho Santa Margarita, CA, USA). Another thoracostomy was created at varying locations for traction and counter-traction. For patients in whom single-incision VATS was attempted, a utility thoracostomy of 5 cm at 4th or 5th intercostal space was created and also protected with a wound retractor. A scalpel and electrocautery (Harmonic ACE®; Johnson & Johnson, Somerville, NJ, USA) were used for pleural adhesiolysis. The major vascular branches were transected with a stapler (Echelon Flex 45 Endopath; Johnson & Johnson, New Brunswick, NJ, USA) and staples (ECR45W white reloads; Johnson & Johnson, New Brunswick, NJ, USA), while minor branches were ligated with silk, a knot pusher, and clips. The segmental bronchus was closed with staples (TA 30 [4.8]; Johnson & Johnson, New Brunswick, NJ, USA) and reinforced with 4–0 sutures (Maxon; Johnson & Johnson, New Brunswick, NJ, USA) if the procedures were performed using thoracotomy. The segmental bronchus was transected with ECR45D gold reloads in the VATS group.

### Statistics

Patients were divided into groups: those who underwent direct or intended thoracotomy, those who were successfully treated with VATS, and those who were converted to thoracotomy due to difficult pleural adhesiolysis, hilar dissection, or bleeding. Continuous variables between two groups (age, percentage of preoperative positive sputum culture, operative time, blood loss, and length of hospital stay) were compared using Student’s t-test, whereas non-continuous variables (sex, procedures, complication, and treatment result) were compared using a χ^2^ test. Risk factors of conversion from VATS to thoracotomy were investigated using multiple logistic regression analysis. Statistical significance was set at *p*<0.05.

### CUSUM analysis

CUSUM (Cumulative summative) analysis was used to analyze the operative time and blood loss in a series of consecutive procedure to see if the procedures had been proficiently performed. Since the 1970s, this technique has been applied in the medical field to analyze the learning curve. It is a graphical method that detects data trends, which is not discernible in other approaches, by calculating the sequential difference between the raw data and the mean value. Surgeons have adopted it as a form of quality control to monitor their performance and consequently validate a particular skill.

## Results

A total of 121 patients underwent segmentectomy. Of these, 18 patients underwent direct or intended thoracotomy for segmentectomy, which was performed before 2007. After 2007, a total of 103 patients attempted VATS segmentectomy, and 64 successfully underwent VATS segmentectomy, including 53 undegoing MITS and 11 undergoing SITS. The remaining 39 patients were converted to thoracotomy. There was no conversion in the SITS group to MITS or thoracotomy. The distribution of resected segments in each group is shown in Table 2, and upper division trisegmentectomy was the most frequently performed procedure. Patient characteristics and demographics are summarized and analyzed in Table 3, and those successfully undergoing MITS or SITS segmentectomy and those converted to thoracotomy were further analyzed in Table 4. Hemoptysis was the most common surgical indication in patients undergoing segmentectomy, and the difference of surgical indication was significant when the converted thoracotomy group and the VATS group were compared (*p* = 0.010). Despite the operative time not differing significantly between each group, the VATS group had significantly less blood loss than the converted and intended thoracotomy group (*p*<0.001 and *p* = 0.005, respectively). The hospital stay in the VATS group was also significantly shorter than the converted and intended thoracotomy group (*p*<0.001 and *p* = 0.005, respectively). Further analysis among patients who attempted VATS segmentectomy revealed that the MITS group had significantly less blood loss than the converted thoracotomy group (*p* = 0.001), and the MITS group also had a shorter hospital stay than the converted thoracotomy group (*p*<0.001). Although the mean operative time, blood loss, and hospital stay in the SITS group were lower than those in the converted thoracotomy group, no statistical difference was found because the patient number in the SITS group was small. Six patients had preoperative positive sputum culture in the intended thoracotomy group. Seven patients have preoperative positive sputum culture in the converted thoracotomy and MITS groups, respectively. Although no disease relapse occurred in the MITS and SITS groups, three patients in the intended thoracotomy group and eight patients in the thoracotomy group had disease relapse.

**Table 2:** The distribution of resected segments in the intended thoracotomy group, converted thoracotomy group, multi-incision thoracoscopic segmentectomy (MITS) group, and single-incision thoracoscopic segmentectomy (SITS) group.

Segment resected	Intended thoracotomy (N = 18)	Converted thoracotomy (N = 39)	MITS (N = 53)	SITS (N = 11)
***Left upper lobe***				
Upper division	2	12	12	2
Ligular	0	2	12	0
Apicoposterior	2	4	1	1
Posterior	0	1	1	1
***Left lower lobe***				
Superior	4	8	5	0
Basal	0	0	3	0
***Right upper lobe***				
Posterior	3	4	8	1
Apical	4	5	6	3
Apicoposterior	4	5	0	3
***Right middle lobe***				
Medial	0	0	2	0
Lateral	0	0	1	0
***Right lower lobe***				
Superior	0	3	3	0

**Table 3:** Demographics of patients undergoing intended thoracotomy segmentectomy, converted thoracotomy segmentectomy, and video-assisted thoracoscopic segmentectomy (VATS), including multi-incision thoracoscopic segmentectomy (MITS), and single-incision thoracoscopic segmentectomy (SITS).

	Intended thoracotomy	Converted thoracotomy	VATS (MITS+SITS)		P value	
	(N = 18)	(N = 39)	(N = 64)	Intended vs. Converted thoracotomy	Converted thoracotomy vs. VATS (MITS+SITS)	Intended thoracotomy vs.VATS (MITS+SITS)
Sex (M, %)	5 (27.8%)	26 (66.7%)	37 (57.8%)	0.675	0.371	0.268
Age, year (mean ± SD)	60.50 ± 8.60	52.56 ± 14.16	51.41 ± 13.04	0.033	0.673	0.007
Indications				0.588	0.010	0.527
Hemoptysis	10	27	24			
Drug-resistance	2	4	6			
Cavitation/Destroyed lung	3	5	22			
Persistent lung lesion	3	3	12			
Segmentectomy in the Same/different lobe	16/2	35/4	64/1	0.922	0.067	0.120
Concomitant wedge resection	2	5	11	0.855	0.553	0.723
Preop positive sputum culture, N (%)	6 (33.3%)	7 (17.9%)	7 (10.9%)	0.198	0.314	0.022
Image characteristic grading (0/1/2)						
Pleura thickening	7/0/1	0/27/12	25/37/2	< 0.001	< 0.001	0.887
Peribronchial lymph node calcification	15/3/0	24/11/4	60/4/0	0.186	< 0.001	0.175
Aspergilloma	13/4/1	27/9/3	51/9/4	0.951	0.461	0.704
Cavity	3/14/1	8/25/6	31/23/10	0.497	0.01	0.007
Tuberculoma	4/8/6	6/19/14	26/21/17	0.819	0.027	0.356
Atelectasis	15/3/0	34/5/0	56/8/0	0.698	0.962	0.699
Bronchiectasis	6/11/1	11/22/6	24/30/10	0.510	0.591	0.428
Bulla	16/2/0	34/4/1	59/4/1	0.781	0.707	0.687
OP time, min (mean ± SD)	153.2 ± 56.3	185.1 ± 67.7	181.5 ± 71.0	0.088	0.803	0.123
Blood loss, mL (mean ± SD)	402.8 ± 626.0	442.8 ± 561.4	140.6 ± 208.7	0.810	< 0.001	0.005
Hospital stay, days (mean ± SD)	12.3 ± 7.6	17.9 ± 19.3	6.1 ± 6.0	0.239	< 0.001	0.001
Complications, N (%)	4 (22.2)	7 (17.9)	5 (7.8)	0.704	0.120	0.084

**Table 4:** Demographics of patients undergoing converted thoracotomy segmentectomy, multi-incision thoracoscopic segmentectomy (MITS), and single-incision thoracoscopic segmentectomy (SITS).

	Converted thoracotomy	MITS	SITS		P value	
	(N = 39)	(N = 53)	(N = 11)	Converted thoracotomy vs. MITS	MITS vs. SITS	Converted thoracotomy vs. SITS
Sex (M, %)	26 (66.7%)	27 (50.9%)	10 (90.9%)	0.132	0.018	0.148
Age, year (mean ± SD)	52.56 ± 14.16	52.09 ± 12.35	48.09 ± 16.25	0.866	0.358	0.375
Indications				0.037	0.069	0.002
Hemoptysis	27	23	1			
Drug-resistance	4	5	1			
Cavitation/Destroyed lung	5	15	7			
Persistent lung lesion	3	10	2			
Segmentectomy in the Same/different lobe	35/4	52/1	11/0	0.159	1.000	0.563
Concomitant wedge resection	5	10	1	0.438	0.672	1.000
Preop positive sputum culture, N (%)	7 (17.9%)	7 (13.2%)	0	0.532	0.339	0.324
Image characteristic grading (0/1/2)						
Pleura thickening	0/27/12	23/28/2	2/9/0	<0.001	0.154	0.002
Peribronchial lymph node calcification	24/11/4	49/4/0	11/0/0	0.001	1.000	0.010
Aspergilloma	27/9/3	40/9/4	11/0/0	0.762	0.062	0.031
Cavity	8/25/6	29/15/9	2/8/1	0.001	0.022	0.824
Tuberculoma	6/19/14	23/17/13	3/4/4	0.017	0.567	0.634
Atelectasis	34/5/0	46/7/0	10/1/0	0.957	1.000	1.000
Bronchiectasis	11/22/6	19/24/10	5/6/0	0.572	0.126	0.156
Bulla	34/4/1	48/4/1	11/0/0	0.877	0.373	0.267
OP time, min (mean ± SD)	185.1 ± 67.7	185.1 ± 73.4	164.5 ± 57.8	1.000	0.385	0.363
Blood loss, mL (mean ± SD)	442.8 ± 561.4	149.1 ± 209.7	100 ± 208.6	0.001	0.482	0.054
Hospital stay, days (mean ± SD)	17.9 ± 19.3	5.5 ± 4.4	9.2 ± 10.5	<0.001	0.063	0.159
Complications, N (%)	7 (17.9)	3 (5.7)	2 (18.2)	0.009	0.201	1.000

The image characteristics of the intended thoracotomy, converted thoracotomy, and VATS groups were analyzed. Patients undergoing converted thoracotomy for segmentectomy had significantly higher grading of pleural thickening than those undergoing intended thoracotomy or VATS segmentectomy (*p*<0.001). Besides, patients undergoing converted thoracotomy had significantly higher grading in peribronchial lymph node calcification (*p*<0.001), cavity (*p* = 0.001), and tuberculoma (*p* = 0.027). Notably, the grading of cavity was significantly higher in the VATS group than that in the intended thoracotomy group (*p* = 0.007). The image characteristics of patients successfully undergoing VATS segmentectomy and those converted to thoracotomy were further analyzed. Between the converted thoracotomy and the MITS groups, significant differences were found in the grading of pleural thickening (*p*<0.001), peribronchial lymph node calcification (*p* = 0.001), cavity (*p* = 0.001), and tuberculoma (*p* = 0.017), which were lower in the MITS group, as shown in Table 4. The grading of other image characteristics, such as bullae, aspergilloma, atelectasis, and bronchiectasis were not significantly different between the converted thoracotomy and MITS groups (*p* = 0.877, 0.762, 0.957, and 0.572, respectively; Table 4). Between the converted thoracotomy and SITS groups, patients in the SITS group had significantly lower grading in pleural thickening (*p* = 0.002), peribronchial lymph node calcification (*p* = 0.010), and aspergilloma (*p* = 0.031). No significant difference was found in the grading of cavity, tuberculoma, atelectasis, bronchiectasis, and bulla (*p* = 0.824, 0.634, 1.000, 0.156, and 0.267, respectively; Table 4). A significant difference was only found in the grading of cavity between the MITS and SITS groups (*p* = 0.022). Risk factor analysis using multiple logistic regression displayed that pleural thickening and peribronchial lymph node calcification were significantly associated with conversion from VATS to thoracotomy (Table 5).

**Table 5:** Multiple logistic regression analysis for risk factor of conversion from VATS to thoracotomy.

Variables	Conversion to thoracotomy	
	OR (95% C.I.)	P value
Age	0.995 (0.955–1.037)	0.810
Preop sputum culture	0.805 (0.127–5.091)	0.818
Pleural thickening	19.667 (3.822–101.193)	< 0.001
Peribronchial lymph node calcification	5.329 (1.270–22.357)	0.022
Aspergilloma	0.968 (0.312–3.005)	0.956
Cavity	0.681 (0.240–1.932)	0.471
Tuberculoma	0.873 (0.426–1.791)	0.712
Atelectasis	1.173 (0.275–5.004)	0.829
Bronchiectasis	0.799 (0.360–1.775)	0.582
Bulla	0.767 (0.162–3.639)	0.738

OR: Odds ratio

C.I.: Confidence interval

Three in the intended thoracotomy and eight patients in the thoracotomy group had disease relapse. No disease relapse was observed among patients who successfully underwent MITS or SITS. Table 6 shows the complications in the intended thoracotomy, converted thoracotomy, MITS, and SITS groups. Four patients in the intended thoracotomy group had complications, and those two with multiple organ failure resulted in mortality. In the converted thoracotomy group, five had postoperative bronchopleural fistulas, four of which were managed with thoracoplasty. Acute respiratory distress syndrome (ARDS) developed in one patient in the thoracotomy group, and one had septic shock and multiple organ failure during their hospital stay, which resulted in mortality in both patients. In the MITS group, one patient had postoperative hemoptysis due to bronchial artery bleeding, another had cardiac arrhythmia, and the other had persistent air leak, which were all managed conservatively. One patient with MDRTB, whose sputum culture remained negative postoperatively, died six months after VATS segmentectomy due to drug-induced hepatic failure. Two patients in the SITS group had complications. One patient developed bronchopleural fistula, which was managed with completion lobectomy and was hospitalized for nine days, and another had ischemic stroke postoperatively, and was hospitalized for 40 days.

**Table 6:** Demographics of patients undergoing intended thoracotomy segmentectomy, converted thoracotomy segmentectomy, multi-incision thoracoscopic segmentectomy (MITS), and single-incision thoracoscopic segmentectomy (SITS) with complications.

	Intended thoracotomy (N = 4)	Converted thoracotomy (N = 7)	MITS (N = 3)	SITS (N = 2)
Sex (M, %)	1 (25%)	6 (85.7%)	2 (66.7%)	2 (100%)
Age, year (mean ± SD)	62.75 ± 13.50	60.13 ± 13.66	58.67 ± 14.01	33.50 ± 4.95
Indications				
Hemoptysis	4	5	2	0
Cavitation/destroyed lung	0	1	1	2
Persistent lung lesion	0	1	0	0
Segment resected				
LUL upper division	0	4	0	0
LUL lingular segment	0	0	2	0
LLL superior segment	0	0	0	0
RUL apicoposterior segment	4	2	0	1
RLL superior segment	0	1	0	0
RUL posterior segment	0	0	1	1
OP time	179.25 ± 70.50	249.0 ± 83.7	177.3 ± 10.5	177.0 ± 36.8
Blood loss	1112.5 ± 1125.0	1021.4 ± 1034.4	116.7 ± 202.1	75 ± 106.1
Hospital stay	14.25 ± 16.50	46.7 ±32.0	15.3 ± 11.6	24.5 ± 21.9
Complications				
Pneumonia/ARDS	0	1	0	0
Wound disruption	0	0	0	0
Persistent air leak	2	0	1	0
Multiple organ failure	2	1	0	0
Hemothorax	0	0	0	0
Bronchopleural fistula	0	5	0	1
Bronchial artery bleeding	0	0	1	0
Arrythmia	0	0	1	0
Stroke	0	0	0	1

**Fig 1:**
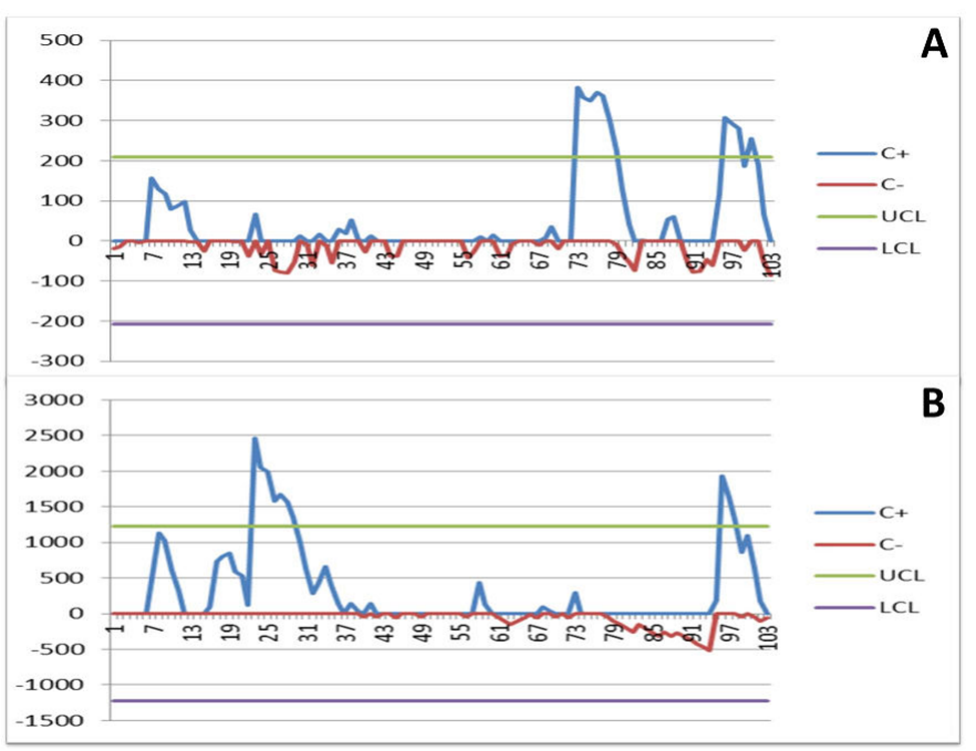
CUSUM chart of operative time (A) and blood loss (B) in patients undergoing intended VATS segmentectomy. The upper control limit (UCL) and lower control limit (LCL) were set as the mean ± 3SD (standard deviation), respectively (Fig 1).

The CUSUM analysis suggests that surgical proficiency of MITS segmentectomy for pulmonary TB can be achieved with approximately 40 cases. However, at the initial stage of performing SITS segmentectomy, based on the experiences of MITS segmentectomy, additional 15 cases were needed to achieve surgical proficiency (Fig 1).

## Discussion

Preservation of pulmonary function with sublobar resection should always be attempted particularly for patients with benign pulmonary disease. We have delineated the efficacy of VATS lobectomy for pulmonary TB [15, 16]. Because we have become experienced in VATS surgery, the main concern of surgical treatment for pulmonary TB is to eradicate lesions, with preservation of as much lung parenchyma as possible. Segmentectomy is technically demanding in patients with pulmonary TB because of the inflammation, adhesion, and destruction, which is even more challenging when VATS is performed. The indication of VATS segmentectomy for pulmonary TB is similar as that for VATS lobectomy [15, 16]. We started VATS anatomic resection for pulmonary TB in 2007 because of the reimbursement. Since Gonzalez-Rivas et al. [17] first described single-incision VATS segmentectomy, or SITS, we started single-incision VATS anatomic resection in 2015, not only for early stage non-small cell lung cancer, but also for benign and inflammatory pulmonary diseases. Our results showed that that MITS and even SITS could be safely performed in selected patients with promising outcomes, and that patients with favorable image characteristics should be attempted with MITS or even SITS because they might have less blood loss and shorter hospital stay.

**Fig 2:**
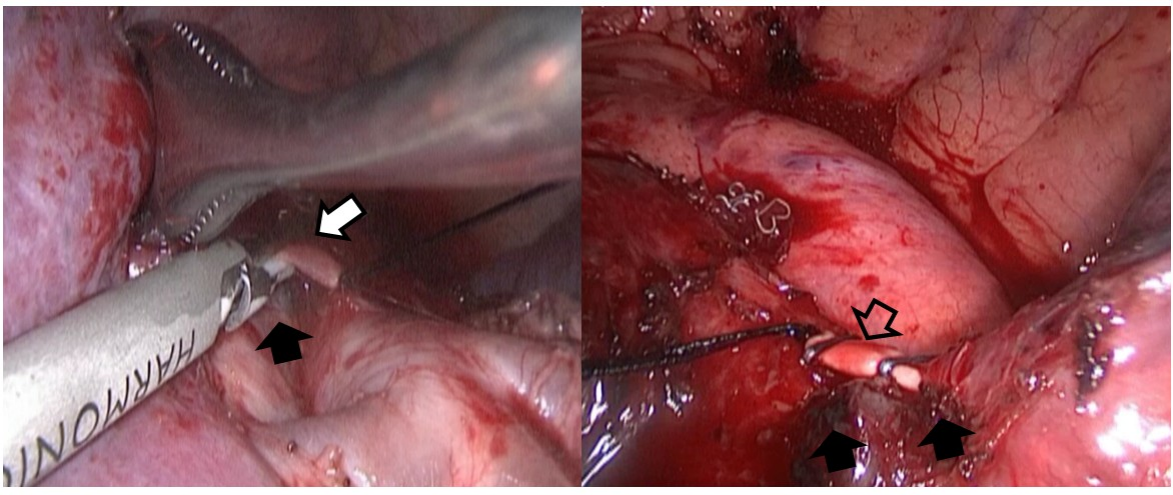
Short segmental arteries with lymph node beside (black arrow), such as the posterior segmental artery of the left upper lobe (white arrow) or the superior segmental artery (empty arrow) were divided with proximal ligation and distal control by ultrasonic scalpel or hemoclip.

**Fig 3:**
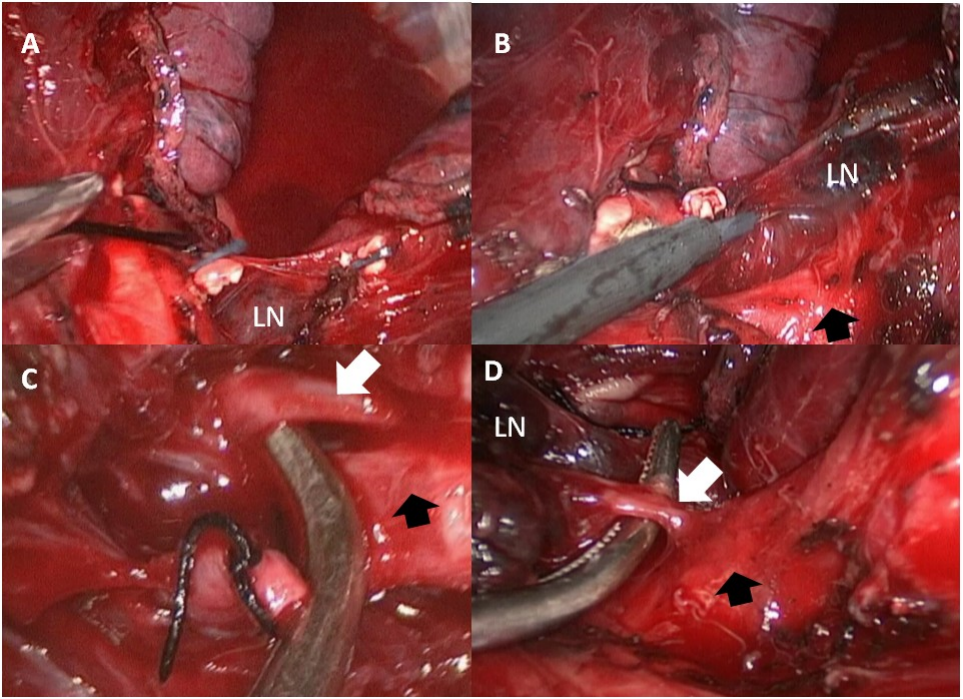
When an adhesive lymph node beside (LN) was noted beside the segmental branch (white arrow) of pulmonary artery (black arrow) (A and B), the segmental branch was dissected more distally and divided it around its branches into the lung (C and D).

Thoracic surgeons willing to perform VATS for pulmonary TB should be familiar with the segmental anatomy of the bilateral upper lobes because most of the lesions are located in the upper lobe. We suggest that the branches of the pulmonary artery be divided first. After the branches of pulmonary vein had been divided, lack of drainage for pulmonary blood flow made bleeding easier, whereas pneumolysis was performed for frequently encountered pleural adhesions. For the upper division of the left upper lobe where segmental arteries were difficult to be accessed before branches of pulmonary veins were divided [18], dividing as many branches as possible avoided oozing and flooding secondary to pneumolysis. D’Amico et al. [14] preferred commencing the segmentectomy from the bronchus. Starting segmentectomy for pulmonary TB from the segmental bronchus, however, is difficult because the bronchus is often surrounded by adhesive lymph nodes and tortuous bronchial artery. Patients with cavitary lesions or traction bronchiectasis tend to have adhesive lymph nodes around the vessels and inflammation of the lung parenchyma, hampering the staple placement, and hence making it difficult and dangerous for stapled division. For short segmental arteries, ligating these vessels by silk knots and reinforcing the proximal stump with a hemoclip is strongly recommended instead of stapling. As reported in the literature [19], the distal stump of artery could be controlled by clipping or with ultrasonic scalpel (Fig 2). In cases, wherein the segmental artery was densely adhered to the lymph nodes and peribronchial tissues, we isolated the segmental artery more distally and divided it around its branches into the lung where the adhesion was less intense (Fig 3). In contrast to the report by Yang et al. [20], which described that the posterior ascending artery of the right upper lobe was the most challenging vessel, we found that the upper division of the left upper lobe was the most difficult segment to be resected in patients with pulmonary TB. Conversion to thoracotomy occurred more often during the early years when VATS segmentectomy was attempted, because most patients deemed for surgical intervention were medicated long term and the inflammation and adhesion were intense. Since surgeons and physicians became experienced and familiar with VATS anatomic resection, early referral and hence early surgical intervention without prolonged medication facilitate the thoracoscopic approach and increase the number of successfully performed VATS anatomic resection, including segmentectomy [21]. We started SITS in 2015 and patients undergoing SITS segmentectomy were relatively short term medicated. As a result the inflammation and adhesion were mild and there was no conversion from SITS to MITS.

**Fig 4:**
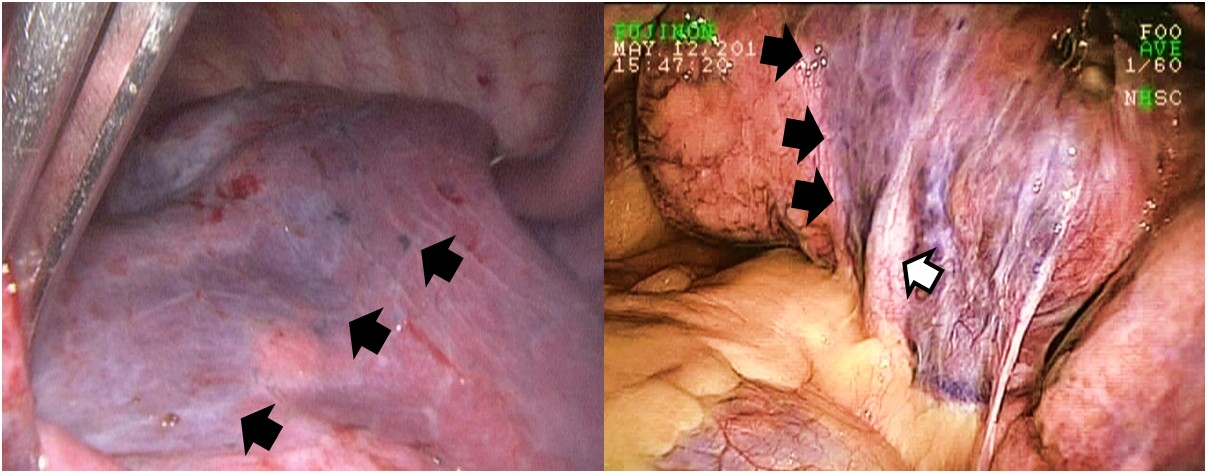
Demarcation of the intersegmental plane (black arrow) resulted from consolidation secondary to TB involvement of the lingular segment as indicated by the lingular vein (white arrow).

Parenchymal separation after vessel control is another pivotal point in VATS for pulmonary TB. The demarcation of the segment to be resected was easily visualized because of the difference in inflammation and parenchymal destruction (Fig 4). Although parenchymal division with electrocautery was reported as safe and feasible in VATS for lung cancer, we preferred stapling parenchymal to minimize the risk of air leak and spillage of infectious material from the diseased segment. Notably, the staple line should be placed over the healthy segment, and the parenchyma squeezed for fifteen seconds before the cartridge was launched for stapling. The balance between tissue tension and compression force must be reached to obtain the perfectly aligned B-shaped staples [22]. A sufficient amount of precompression time, therefore, should be waited to secure staple formation [23]. We routinely reinforced the staple line leakage, if any, with hand-sewing stitches to minimize the risk of air leakage. One disadvantage of stapled parenchymal division was that the staple opening was not wide enough, hence, that the number of cartridge was usually more than expected. The reimbursement of National Health Administration for VATS encouraged thoracic surgeons to further its application from malignant to infectious disease, conveying the benefit of minimally invasive surgery to patients with pulmonary TB, particularly those who were often socioeconomically compromised. Therefore, patients undergoing VATS therapeutic resection would not have been otherwise deprived of the chance of cure because of the cost of surgery.

Moving forward from MITS to SITS requires more effort and is achievable, which was revealed on the CUSUM chart that it took approximately additional 15 cases to achieve surgical proficiency. Dissection is initiated around the hilar structure with the pleural adhesion, if any, left behind as traction or counter-traction. Immediately after the hilar structures had been ligated and separated, the pleural adhesion could be easily managed without much bleeding. The pleural adhesion and management during the early years of VATS for pulmonary TB, interestingly, not only resulted in the increase in operative blood loss as revealed in the CUSUM chart, but conversion to thoracotomy as well. Nonetheless, one commonly encountered technical problem is the fencing of instruments at the incision, which increased the operative time but not the blood loss at the initial stage of performing SITS. Adjustment for different instrument alignments at the incision usually works well to overcome this problem. Based on the results on the CUSUM chart, initiation with a larger incision is recommended for surgeons willing to step further from MITS to SITS. The parallel direction instead of a certain angle between the thoracoscope and instruments has also been reported to facilitate the dissection [24, 25]. With the improvement of the various instruments specifically designed for SITS, the ease and readiness of SITS for pulmonary TB could be highly anticipated.

The comparison between the thoracotomy, MITS, and SITS groups could be invalid because of different disease extent. Patients undergoing successful MITS or SITS had lower grading in pleural thickening and peribronchial lymph node calcification than in those converted to thoracotomy. In fact, extensive pleural adhesion and peribronchial lymph node calcification highly indicated conversion to thoracotomy. Granulomatous involvement and resultant tuberculoma and calcification cause severe adhesion between the lymph nodes and the vascular and bronchial structure, combined with distortion of the anatomy. The presence of a chronically thickened cavity wall with obliterated pleural space in a damaged lung should be treated with cavernostomy and thoracoplasty or cavernostomy with intrathoracic muscle flap transposition instead of VATS [26]. We previously reported that the presence of severe pleural adhesion and multiple cavities significantly increased the surgical risk during pulmonary resection for pulmonary TB [15, 27]. Notably, although a variable degree of pleural adhesion was encountered, the amount of blood loss and risk of complications significantly decreased because the dissection was facilitated by the sharp vision of the VATS system. Because patients undergoing segmentectomy instead of lobectomy were enrolled, the extent of the cavity was generally limited. The presence of a simple cavity, however, seemed to facilitate the accomplishment of SITS because the pleural adhesion might have served as traction or counter-traction. This was evidenced by the comparison between the intended thoracotomy and the VATS group. The grading of cavity was significantly higher in the VATS group than the intended thoracotomy group, and the blood loss and hospital stay were significantly decreased. Risk factors of conversion to thoracotomy included pleural thickening and peribronchial lymph node calcification but not cavity. With the exclusion of the aforementioned lesions mandating a thoracotomy, more liberal use and initial attempt of MITS or even SITS should be advocated as an adjunct in the treatment for pulmonary TB.

Patients with medical treatment failure for TB, MDRTB, and non-tuberulous mycobacterium (NTM) are at substantial risk for complication after anatomic resection because endobronchial colonization has been reported as a risk factor for postoperative bronchopleural fistula [28]. We did not reinforce patients undergoing segmentectomy, although the bronchial stump has been known to be reinforced with a viable intercostal muscle, pleural flaps, or pericardial fat pad [29, 30]. Because bronchial stump healing is associated with peribronchial lymph node or soft tissue coverage, we believe that avoidance of overt dissection around the bronchus, which is frequently performed in pulmonary diseases of chronic inflammation and adhesion, preserves the healing capacity of the bronchial stump. Stapled closure was successful in the MITS and SITS groups, which could be attributed to the favorable image characteristics and minor extent of inflammation and adhesion. We found that the image characteristics in the MITS or SITS group have minor pleural thickening and peribronchial lymph node calcification, which is in concordance with our previous publication [15]. Interestingly, besides the preoperatively positive sputum culture, patients with surgical complications had image characteristics of higher grading. The emergence of prolonged medical treatment indicated relentless inflammation and parenchymal destruction that the extent of pleural thickening, peribronchial lymph node calcification, the amount of operative blood loss, and length of hospital stay significantly increased in the thoracotomy group. Besides those aforementioned techniques, successful surgeries also rely on early referral after medical treatment failure, proper selection of patients, and complete anti-TB drug treatment under surveillance. Although eight patients in the thoracotomy group had disease relapse, the patient number was small, and the follow-up period was short. A larger patient population and long-term follow up are required before we reach a solid conclusion that MITS or SITS is efficient for patients with TB with medical treatment failure.

Some limitations exist in this study because it was retrospective and not randomized. We started using VATS at the beginning of 2007, and moved forward from MITS to SITS in 2015. Before the learning curve of VATS was overcome, some suitable patients might have been excluded. The number of patients successfully undergoing SITS was limited. Although there was no conversion from SITS to MITS or thoracotomy, which could be attributed for the early referral and hence short term medication, further validation of its feasibility and efficacy should be conducted. With increased experiences in performing VATS, the surgeon’s tolerance and perseverance for performing dissection over adhesive and chronically inflammatory areas may gradually increase, and more patients with pulmonary TB will benefit from VATS. The postoperative care of thoracic surgical patients has become more delicate in recent years. Directly observed treatment strategy of tuberculosis started since 2007 in our country, and the patients with MDRTB were treated under the surveillance by the center of disease control since 2008. Therefore, patients received better disease control and patient care in the era of segmentectomy performed by VATS, improving their surgical results. Pulmonary function test was not routinely performed in patients undergoing thoracic surgery before 2007. Pulmonary function test was performed in only 11 of 34 patients because they had relatively poor performance. In the VATS era, all patients except those who had medical treatment failure underwent standard pulmonary function test. Thus, the difference of pulmonary function between these two patient groups might be overestimated.

## Conclusion

Our results show that VATS segmentectomy, including MITS and SITS for pulmonary TB is safe and feasible in patients with favorable image characteristics, providing the benefits of minimally invasive surgery and preserving the lung parenchyma. For patients with pulmonary TB deemed for anatomic resection, MITS or even SITS could be attempted, and early referral might facilitate the use of MITS or SITS. Further investigation is required for patients with medical treatment failure.

## Supporting information

S1 File Characteristics of patients undergoing surgery for pulmonary tuberculosis. (SAV)
